# Mental health disorders and alcohol use are associated with increased likelihood of smoking relapse among people living with HIV attending routine clinical care

**DOI:** 10.1186/s12889-019-7705-1

**Published:** 2019-10-29

**Authors:** Cosmas M. Zyambo, Greer A. Burkholder, Karen L. Cropsey, James H. Willig, Craig M. Wilson, C. Ann Gakumo, Andrew O. Westfall, Peter S. Hendricks

**Affiliations:** 1 Department of Health Behavior, School of Public Health, University of Alabama at Birmingham, Birmingham, USA; 2 Division of Infectious Diseases, School of Medicine, University of Alabama at Birmingham, Birmingham, USA; 3 Department; Epidemiology of Microbial Diseases, School of Public Health, Yale University, New Haven, USA; 4 Department of Community and Family medicine, School of Public Health, University of Zambia, Lusaka, Zambia; 5 Department of Psychiatry, School of Medicine, University of Alabama at Birmingham, Birmingham, USA; 6 Department of Epidemiology, School of Public Health, University of Alabama at Birmingham, Birmingham, USA; 7 Department of Nursing, University of Massachusetts, Boston, MA USA; 8 Department of Biostatistics, School of Public Health, University of Alabama at Birmingham, Birmingham, USA

**Keywords:** HIV, PLWH, Smoking cessation, Relapse, Anxiety, Depression, Alcohol use, Mental health disorders

## Abstract

**Background:**

People living with HIV (PLWH) have a high level of interest in quitting smoking, but only a small proportion have sustainable abstinence 6 months after cessation. Few investigations have focused on relapse to smoking among PLWH. In this investigation, we evaluated the prevalence of relapse after smoking cessation and the characteristics associated with smoking relapse using a retrospective, longitudinal cohort of PLWH during an eight-year observation.

**Methods:**

All patients aged ≥19 years that reported current smoking during the study period and then reported not smoking on a subsequent tobacco use questionnaire (quitters) were eligible for the study. In addition, patients required at least one subsequent follow-up visit after quitting where smoking status was again reported to allow for assessment of relapse. A Cox proportional hazard model was fit to evaluate factors associated with smoking relapse in PLWH attending routine clinical care.

**Results:**

Of the 473 patients who quit smoking in the study, 51% relapsed. In multivariable analysis, factors significantly associated with a higher likelihood of relapse were anxiety symptoms (HR = 1.55, 95% CI [1.11, 2.17]) and at-risk alcohol use (HR = 1.74, 95% CI [1.06, 2.85]), whereas antiretroviral therapy (ART) adherence (HR = 0.65, 95% CI [0.49, 0.99]) and longer time in care (HR = 0.94, 95% CI [0.91, 0.98]) were associated with a reduced likelihood of relapse after cessation.

**Conclusion:**

Our study underscores the high prevalence of smoking relapse that exists among PLWH after they quit smoking. Successful engagement in mental health care may enhance efforts to reduce relapse in the underserved populations of PLWH.

## Background

Tobacco smoking is one of the most significant public health threats that the world has ever faced, currently claiming the lives of seven million people annually [1], and 480,000 people annually in the United States (US) alone [2, 3]. Recent studies report smoking prevalence estimates among people living with HIV (PLWH) in the US ranging from approximately 39 to 70% [4–6]-two to three times higher than the general population [7–9]. Most PLWH are aware of the health risks associated with smoking, and though a high level of interest in quitting exists [10–12], only a small proportion have sustained abstinence 6 months after cessation [13]. In the general population, 68% of the smokers would like to quit, and at least 57.2% report attempting to quit in the past year [14]. However, the smoking prevalence has not substantially declined over recent decades, and the quit rate has remained as low as 7.4% [14]. In addition, many of those who manage to quit often relapse [15–18]. The addictive nature of cigarettes including their associated withdrawal symptoms, such as anxiety, depression, and weight gain [19, 20], may require multiple quit attempts [21, 22] to break the cycle of remission and relapse [23, 24].

PLWH who are smokers have a high prevalence of mental health disorders and illicit drug use, as well as limited economic resources and diminished access to care. These characteristics collectively place them at a high risk for relapse [11, 25–27] . Thus, there is a need not only to support initial smoking cessation among PLWH but also to have interventions that monitor for and help prevent smoking relapse. Although studies on factors relating to smoking relapse have been conducted, most of them have been conducted in the general population [16, 17, 28, 29]. There is a paucity of studies on smoking relapse that have targeted special populations such as PLWH [18], and none evaluating factors related to smoking relapse among PLWH in the US. Therefore, we used a sample from a large cohort of PLWH engaged in routine clinical care to report the prevalence of smoking relapse as well as the sociodemographic, clinical, behavioral, and psychological factors associated with smoking relapse.

## Materials and methods

### Setting and population

The University of Alabama in Birmingham (UAB) 1917 HIV Clinic cohort is an ongoing longitudinal HIV clinical cohort protocol which was established in 1992 (http://www.uab.edu/medicine/1917cliniccohort/) and has been well-described in our previous work [30]. Our cohort’s clinical electronic database captures extensive sociodemographic, clinical, behavioral, and psychological data on all the PLWH receiving primary care and subspecialty care at the UAB HIV/AIDS clinic (1917 Clinic). This retrospective longitudinal study utilized data on PLWH seen at the 1917 Clinic between April 2008 and April 2017.

### Sources of data

Electronic query using MS SQL Server 2008 was used to obtain sociodemographic information, comorbidities, visit information and clinically relevant laboratory results from the UAB 1917 HIV Clinic electronic medical records (EMR) and administrative databases. Behavioral and psychological information from PLWH receiving care at the clinic are captured electronically by a patient-reported outcome (PRO) system. Patients complete computerized, standardized, validated questionnaires that are self-administered every 4–6 months during a routine visit and take 10–15 min. Using the PRO tobacco use questionnaire, patients were asked, “Have you smoked more than 20 cigarettes in your lifetime?” If the answer was “Yes,” then the follow-up question was, “Do you currently smoke cigarettes?”. The PRO system also contains questionnaires regarding depression (Patient Health Questionnaire-9 [PHQ-9]); anxiety (PHQ-Anxiety); for alcohol use (the Alcohol Use Disorder Identification Test - Consumption [AUDIT-C]); substance use (the Alcohol, Smoking, and Substance Involvement Screening Test –[ASSIST] and antiretroviral therapy (ART) adherence, (the Adult AIDS Clinical Trials Group [AACTG] Adherence Instrument) [31]. Data was de-identified before the analyses.

### Eligibility criteria

**Fig. 1:**
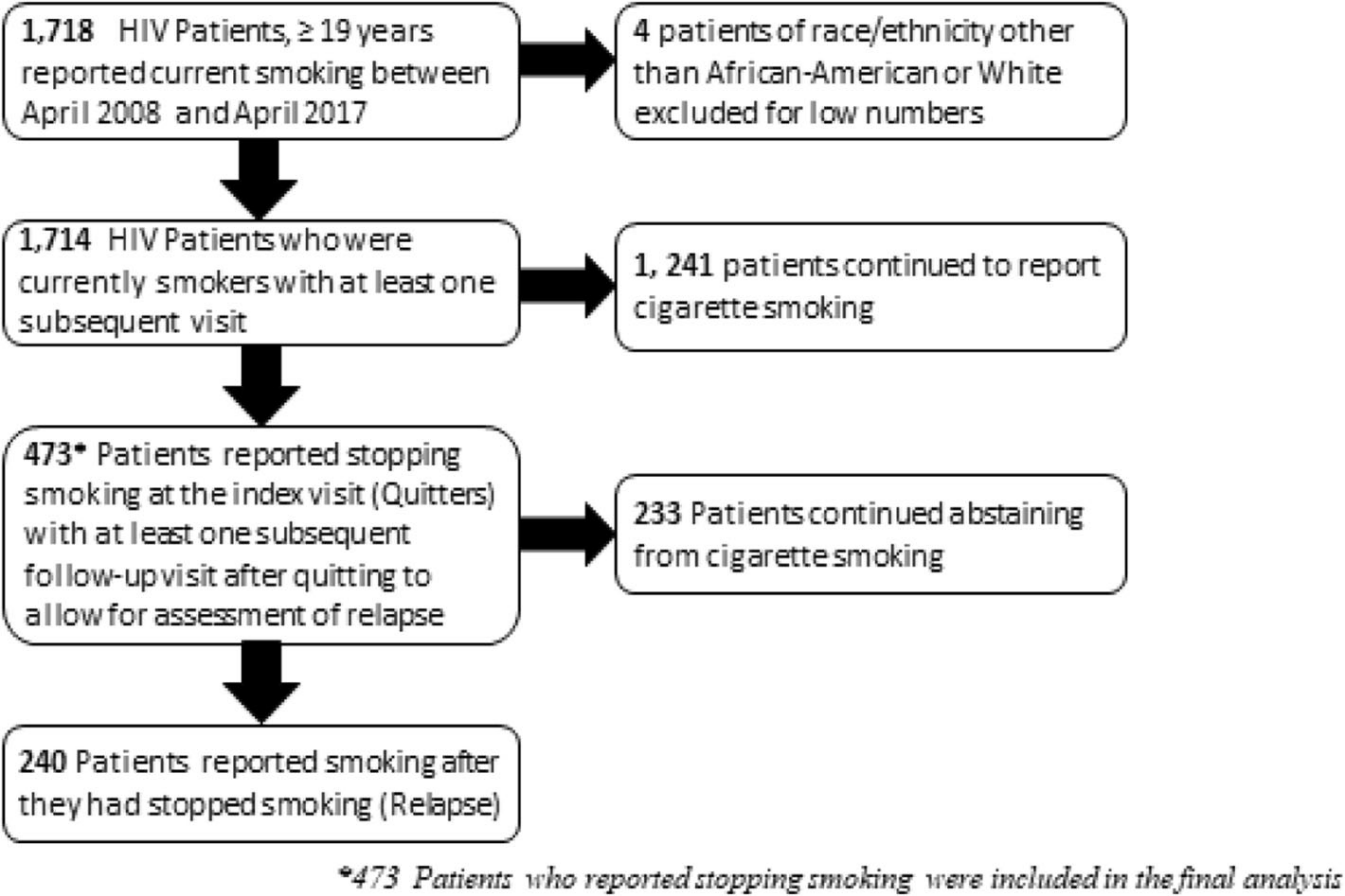
Flow diagram of criteria for inclusion in analysis of smoking relapse among HIV- infected patients attending the UAB 1917 HIV clinic between April 2008 and April 2017

All 1917 Clinic patients aged ≥19 years (Age of consent in Alabama) who reported current smoking between April 2008 and April 2017 and then reported not smoking on a subsequent tobacco use questionnaire (quitters) were eligible for the study. In addition, patients required at least one subsequent follow-up visit after quitting where smoking status was again reported to allow for assessment of relapse (Fig. 1). The index visit in this study was defined as the first primary care visit where a patient reported no longer currently smoking on the PRO. The final analysis included 473 PLWH who quit smoking.

### Primary outcome and variables

The primary outcome was the time to smoking relapse after quitting. Time to relapse was defined as the number of months from the index date until the first follow-up visit where current smoking was reported. Individuals who did not relapse were censored at the date of their last follow-up PRO in the study period where smoking status was reported. Patients were also censored when they died or were lost to follow-up (not being in care for > 12 months). Independent variables were similar to those used in our prior work on smokers in this cohort [30] and included: 1) *Sociodemographic characteristics—*age, composite gender/sexual orientation (men who have sex with men (MSM), heterosexual men, and women), and race/ethnicity. Only African-American and White patients were included. We excluded 4 people (0.8%) of other race /ethnicity due to low sample size. 2) *Clinical characteristics—*years in care at the 1917 HIV clinic, type of health insurance (private, public, or uninsured), non-adherence to ART (defined as missing ≥1 dose in the last 7 days using the AACTG Adherence Instrument), history of smoking related co-morbidities including respiratory diseases (asthma, chronic obstructive pulmonary disease [COPD], and bacterial pneumonia), metabolic diseases (diabetes and dyslipidemia), and cardiovascular diseases (stroke, myocardial infarction, coronary heart disease [CHD] and hypertension), history of any cancer, and HIV-related laboratory values (plasma HIV-1 RNA [viral load; VL] and CD4 count). 3) *Behavioral and psychological characteristics —* symptoms of major depression (based on a PHQ-9 score ≥ 10); anxiety symptoms (based on PHQ Anxiety score ≥ 10); substance use (non-prescription use of prescription opioids or use of illicit opioids, cannabis, crack/cocaine, amphetamines, sedatives, inhalants, or hallucinogens indicated on the ASSIST) and alcohol use as per the AUDIT-C (at-risk score defined as ≥5 for men and ≥ 4 for women). These variables were used as a single baseline value at index visit. History of comorbidity (respiratory, metabolic, or cardiovascular disease or any cancer) was defined by presence of that comorbidity on a patient’s problem list in the EMR on or prior to the index visit. For time-varying variables, the value closest to the index visit was used.

### Statistical analysis

We calculated the proportion of quitters who relapsed versus those who did not, overall and stratified by patient characteristics. Categorical variables are reported as frequencies with percentages and continuous variables are reported as means (standard deviation [SD]). Chi square tests were used to compare categorical variables. Kaplan-Meier curves were constructed to evaluate the time to smoking relapse. Cox proportional hazards models producing unadjusted and adjusted hazard ratios (HRs) and the corresponding 95% confidence intervals (CIs) were used to assess associations between the independent variables and smoking relapse. Clinically relevant variables were determined a priori and included in the full model regardless of the statistical significance in the univariate regression model (age, gender/sexual orientation, race/ethnicity, comorbidities, CD4 count and plasma HIV-1 RNA). Additional variables were included in the full model based on univariate statistical significance (*p* < 0.25) with smoking relapse (major depression, anxiety symptoms, substance use, and alcohol use). Statistical significance in the adjusted model was set at two-sided 0.05 level. Analysis was performed using IBM SPSS Statistics for Windows, Version 24.0 (IBM Corp., Armonk, N.Y., USA).

## Results

Among the 473 PLWH who quit smoking during the study period (Fig. 1), the mean age (± SD) was 46.7 (11.2) years. Approximately half (50.7%) of the population was African-American and most were male (83.8%). (Table 1). Over half the patients were insured (public 26.6%, private 32.1%, and uninsured 41.2%). In terms of comorbidities, 14.8% of the patients had cardiovascular diseases, 21.4% had respiratory diseases, 15.0% had metabolic diseases, and 7.2% had history of any cancer. The VL was suppressed (< 200 copies/ml) in 81.6% of the patients, and the CD4 cell count was ≥200 cells/μL in 89.6%. Major depressive symptoms and anxiety symptoms were common (20.9 and 28.2% of patients respectively) as was current substance use (35.7%). At-risk alcohol use was reported by 11.8% of patients.

**Table 1:** The Characteristics of HIV+ -Smoking Quitters receiving care at the UAB 1917 HIV/AIDS clinic between April 2008–April 2017 according to Relapse status

	Overall N(473) ^a^ N(%)	Relapse (Yes = 240) ^a^ n(%)	Relapse (No = 233) ^a^ n(%)
Socio-demographic			
Age (years), mean (SD)	46.7 (11.2)	46.4 (11.1)	47.1 (11.4)
Gender/sexual orientation			
Women	75 (16.2)	38 (16.1)	37 (16.3)
Male heterosexual	79 (17.1)	35 (14.8)	44 (19.4)
MSM	309 (66.7)	163 (69.1)	146 (64.3)
Race/ethnicity			
White	231 (49.3)	120 (50.2)	111 (48.3)
African American	238 (50.7)	119 (49.8)	119 (51.7)
Clinical/medical			
Length in care (years) mean (SD)	8.8 (4.74)	9.14 (4.62)	8.54 (4.85)
Adherent to ART	66 (15.6)	34 (16.4)	32 (14.7)
Health Insurance			
Private	152 (32.1)	78 (36.2)	74 (31.8)
Public	126 (26.6)	64 (26.7)	62 (26.6)
Uninsured	195 (41.2)	98 (40.8)	97 (41.6)
Comorbidities^b^			
Cardiovascular	70 (14.8)	30 (12.5)	40 (17.2)
Respiratory	101 (21.4)	59 (24.6)	42 (18.0)
Metabolic	71 (15.0)	32 (13.3)	39(16.7)
Cancer	34 (7.2)	21 (8.80)	13 (5.60)
Laboratory parameter^c^			
Plasma HIV-1 RNA < 200 copies/ml	386 (81.6)	190 (79.2)	196 (84.1)
CD4 count (cells/μL)			
< 200	49 (10.4)	20 (8.40)	29 (12.4)
≥ 200	423 (89.6)	219 (91.6)	204 (87.6)
Behavioral and psychological^e^			
Depressive symptoms	97 (20.9)	58 (24.6)	39 (17.0)
Anxiety symptoms	130 (28.2)	80 (34.2)	50 (22.0)
Substance abuse^d^			
Never	65 (13.7)	33 (13.7)	32 (13.7)
Prior	177 (37.4)	91 (37.9)	86 (36.9)
Current	49 (10.4)	28 (11.7)	21 (9.01)
Unknown	182 (38.5)	88 (36.7)	94 (40.3)
Alcohol abuse			
No risk	150 (34.6)	74 (33.6)	136 (65.1)
Low risk	232 (53.6)	111 (50.5)	121 (56.8)
At risk	51 (11.8)	35 (15.9)	16 (7.5)

Abbreviations: *ART* antiretroviral therapy, *HIV* human immunodeficiency virus, *MSM* men who have sex with men, *SD* standard deviation, *UAB* University of Alabama at Birmingham Missing data: Race, 0.8%; Gender/sexual orientation, 2.1%; Adherence, 10.4%; CD4 count, 0.2%; Depression (PHQ-9), 1.7%; Anxiety (PHQ-A), 2.5%; Alcohol abuse, 8.5%;

^a^Column percentages; ^b^Comorbidities: respiratory (asthma, chronic obstructive pulmonary disease, bacterial pneumonia); metabolic (diabetes and dyslipidemia); cardiovascular (stroke, myocardial infarction, coronary artery disease, hypertension); ^c^Lab value closest to index visit; ^d^Substance abuse includes street opioids, prescription opioids, marijuana, crack/cocaine, amphetamines, sedatives, inhalants, hallucinogens; ^e^At baseline quit value

Plasma HIV-1 RNA < 200 copies/ml, Depressive symptoms, Anxiety symptoms, Alcohol abuse were significant at *p* value < 0.05 level

**Fig. 2:**
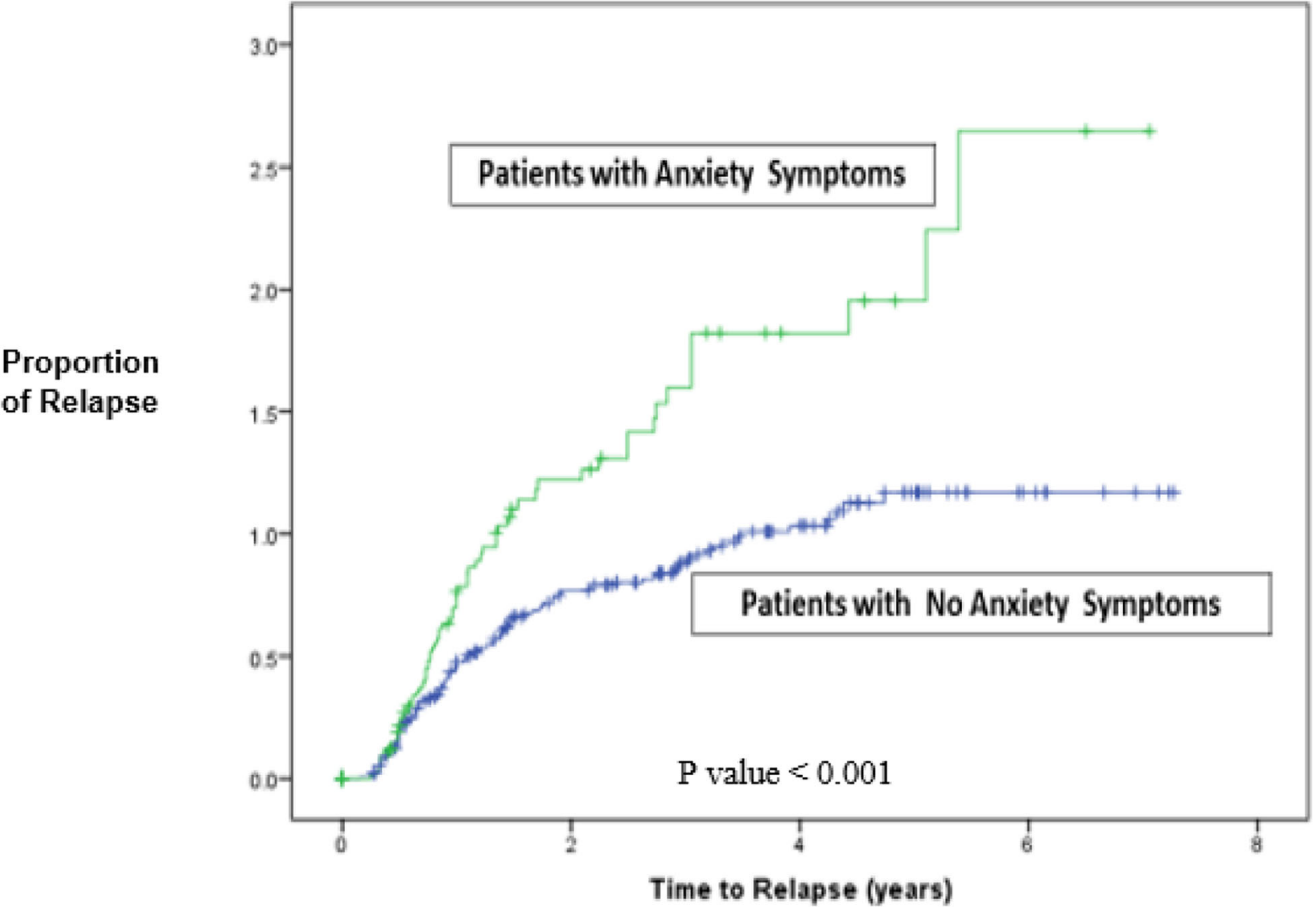
Time to smoking relapse for PLWH at the UAB 1917 Clinic (*N* = 473) with anxiety symptoms versus those without anxiety symptoms. *P* value < 0.001

Overall, 51% of quitters relapsed after cessation. Among patients with major depressive symptoms, 59.8% of the patients relapsed and among those with anxiety symptoms 61.5% relapsed. The median follow-up time for patients with anxiety symptoms was 0.96 years (vs. 1.73 years for patients with no anxiety symptoms) (Fig. 2). In univariate analysis, older age, greater length of time in care, having a metabolic comorbidity, and adherence to ART were significantly associated with decreased likelihood of relapse whereas having public as compared to private insurance, major depressive symptoms, anxiety symptoms, and at-risk alcohol use were significantly associated with increased likelihood of relapse. (Table 2). In the multivariable analysis, factors significantly associated with a reduced likelihood of relapse included adherence to ART (HR = 0.65, 95% CI [0.49, 0.99]) and longer time in care (HR = 0.94, 95% CI [0.91, 0.98]), whereas patients with anxiety symptoms (HR = 1.55, 95% CI [1.11, 2.17]) and those with at-risk alcohol use (HR = 1.74, 95% CI [1.06, 2.85]) were more likely to relapse. Patients with major depression were more likely to relapse (HR = 1.48, 95% CI [0.99, 2.19]) but this difference did not reach statistical significance in the adjusted model.

**Table 2:** Cox proportional hazard model examining factors associated with relapsing among HIV+ smokers receiving care at the UAB 1917 HIV/AIDS clinic between April 2008 and April 2017

	Relapse			
	Unadjusted HR (95% CI)	*p*-value	Adjusted HR (95% CI)	*P*-value
Socio-demographic				
Log age10^**a**^	**0.29 (0.09, 0.92)**	**0.03**	0.57 (0.09, 3.48)	0.56
Gender/sexual orientation				
Women	REF		REF	
Male heterosexual	0.85 (0.54, 1.34)	0.48	0.97 (0.56, 1.68)	0.91
MSM	1.09 (0.77, 1.56)	0.62	1.21 (0.79, 1.84)	0.38
Race/ethnicity				
White	REF		REF	
African American	1.09 (0.85, 1.42)	0.42	1.06 (0.76, 1.48)	0.73
Clinical/medical				
Length in care	**0.95 (0.92, 0.98)**	**0.00**	**0.94 (0.91, 0.98)**	**0.00**
Adherent to ART^**e**^	**0.63 (0.44, 0.92)**	**0.02**	**0.65 (0.42, 0.99)**	**0.04**
Health Insurance^**e**^				
Private	REF		REF	
Public	**1.41 (1.01, 1.96)**	**0.04**	1.46 (0.97, 2.19)	0.06
Uninsured	1.24 (0.92, 1.67)	0.16	0.86 (0.59, 1.25)	0.44
Comorbidities^**b**^				
Cardiovascular	0.72 (0.49, 1.06)	0.09	0.87 (0.54, 1.40)	0.56
Respiratory	1.05 (0.78, 1.41)	0.78	1.23 (0.86, 1.78)	0.24
Metabolic	**0.59 (0.40, 0.85)**	**0.00**	0.82 (0.51, 1.32)	0.42
Cancer	0.98 (0.63, 1.55)	0.96	0.99 (0.58, 1.71)	0.98
Laboratory parameter^**c**^				
Plasma HIV-1 RNA < 200 copies/ml	0.96 (0.70, 1.31)	0.77	0.77 (0.56, 1.07)	0.12
CD4 count (cells/μL)				
≥ 200	0.89 (0.56,1.41)	0.62	0.61 (0.35, 1.07)	0.09
Behavioral and psychological^e^				
Major Depression	**1.57 (1.16, 2.11)**	**0.00**	1.48 (0.99, 2.19)	0.053
Anxiety symptoms	**1.68 (1.28, 2.21)**	**0.00**	**1.55 (1.11, 2.17)**	**0.01**
Substance abuse^d^				
Never	REF		REF	
Prior	1.20 (0.89, 1.61)	0.23	1.48 (0.91, 2.42)	0.12
Current	1.19 (0.77, 1.82)	0.43	1.35 (0.94, 1.94)	0.10
Unknown	1.46 (0.97, 2.18)	0.06	1.02 (0.61, 1.64)	0.99
Alcohol abuse				
No risk	REF		REF	
Low risk	1.01 (0.75, 1.35)	0.96	1.15 (0.81, 1.65)	0.44
At risk	**1.69 (1.13, 2.53)**	**0.01**	**1.74 (1.06, 2.85)**	**0.03**

Abbreviations: *AIDS* acquired immunodeficiency syndrome, *ART* antiretroviral therapy, *CI* confidence interval, *HIV* human immunodeficiency virus, *MSM* men who have sex with men, *UAB* University of Alabama, Birmingham; clinically relevant variables with *P* values < 0.25 were included in the adjusted model. Bold typeface indicates statistical significance at the *P* < 0.05 level

^a^Hazard ratio per 10-year increment; ^b^Comorbidities: cardiovascular (stroke, myocardial infarction, cardiovascular disease, hypertension); respiratory (asthma, chronic obstructive pulmonary disease, bacterial pneumonia); metabolic (diabetes and dyslipidemia), patients without comorbidities were used as the reference group; ^c^Lab value closest to index visit; ^d^Substance abuse includes street opioids, prescription opioids, marijuana, crack/cocaine, amphetamines, sedatives, inhalants, hallucinogens; ^e^At baseline quit value

## Discussion

This study aimed to estimate the prevalence of smoking relapse and examine the socio-demographic, clinical, behavioral, and psychological characteristics associated with relapse among PLWH engaged in routine clinical care. Overall, half of PLWH relapsed after smoking cessation. Anxiety symptoms and at-risk alcohol use were associated with an increased likelihood of relapse whereas ART adherence and greater length of time in care were associated with a reduced likelihood of relapse.

The percentage of PLWH who relapsed after quitting in the current study (51%) is higher than that reported in the general population (< 25%) [24, 32] and is relatively similar to that in another study among PLWH in the Swiss HIV cohort [18]. It is not unexpected that our relapse estimates are higher than those in the general population, as PLWH who are smokers also have a high prevalence of mental health disorders and substance use, combined with diminished access to care. All these characteristics likely conspire to place them at a higher risk of smoking relapse [11, 25–27]. Indeed, we found that PLWH with anxiety symptoms and at risk alcohol use were more likely to relapse. Those with symptoms of major depression were also more likely to relapse, although this association did not reach statistical significance in the adjusted model, perhaps owing to sample size.

The prevalence of anxiety and depression was higher in our sample than in the general population and in conformity with other studies among PLWH [33, 34]. These psychological symptoms have been shown to predict relapse [35]. Previous studies have shown that anxiety is the most commonly reported smoking withdrawal symptom, affecting almost 87% of the patients who stop smoking [35, 36]. Anxiety increases in the first 1–3 days after smoking cessation and then gradually reduces in intensity [37–39], and has been cited as a robust predictor of relapse. It is not surprising as well in our study that the proportion of those who relapsed was higher in patients with anxiety symptoms than those with no anxiety symptoms. Despite the positive association between depression and relapse not reaching statistical significance in our study, studies in the general population have shown that an increase in depressive symptoms before or after quitting smoking predicts relapse [40, 41] and a recent prospective study reported that depression is associated with smoking relapse [42]. The association between at-risk alcohol use and smoking relapse observed among our patients also is consistent with previous findings in the general population [43–45] and alcohol use has previously been suggested as a potential factor increasing the likelihood for smoking cessation failure among PLWH [46].

Our study found that patients who undergo a longer duration of care and are adherent to ART are less likely to relapse. The training of physicians and clinic staff on the 5As of smoking cessation (Ask, Advise, Assess, Assist and Arrange) could be one plausible explanation for how length in care at the 1917 HIV Clinic was associated with lower rates of relapse. The other plausible explanation is that longer duration of care would likely mean more episodes of smoking cessation counseling. Among our study population 15.6% were non-adherent to ART, which is not unexpected as previous studies have indicated that ART adherence in PLWH is a significant problem [47–49] but more so in PLWH who smoke [50–52]. Aggarwal et al. have reported that smokers are less adherent to other medications as well [53]. Although we did not assess use of smoking cessation medications in this study, adherence to ART is likely a marker of other health behaviors; it is plausible that patients who did not adhere to ART also did not adhere to smoking cessation medication or counseling from providers.

Our study has limitations. First, we used self-reported data in our analyses. However, studies have shown that computer-administered questionnaires have comparable validity to that of clinical interviews [54]. Second, as quit and relapse dates were self-report of current smoking status of the patient at clinic visits, these dates likely do not represent a precise estimation of the primary outcome variable “time to relapse”. Third, although we were able to adjust for a number of patient characteristics with the potential to impact smoking relapse, residual confounders may be present. Fourth, due to the retrospective observational nature of the study design, we can comment on associations but cannot demonstrate causality. Finally, findings from this single study site may not be generalizable to other national and international settings.

A strength of our study is a relatively large sample of well-characterized PLWH engaged in routine clinical care. Routine clinical data often represent a more diverse population than clinical trials due to the absence of enforcing any enrollment criteria and is more reflective of the “real world” experience of clinicians providing HIV care. The application of PROs using standardized, validated instruments to measure behavioral and psychological domains is an additional strength of the current study.

## Conclusion

Our findings underscore the high risk of relapse in PLWH after they quit smoking, particularly among patients who have anxiety symptoms and at-risk alcohol use. The mental health needs and alcohol use of this population will need to be addressed in concert with smoking cessation inventions in order to prevent relapse.

## Data Availability

The de-identified datasets used and /or analyzed during the current study are available at UAB on reasonable request with permission from Dr. Greer A. Burkholder (gburkholder@uabmc.edu).

## Abbreviations

AACTG: Adult AIDS Clinical Trials Group
ART: Antiretroviral therapy
ASSIST: Alcohol, Smoking, and Substance Involvement Screening Test
AUDIT-C: Alcohol Use Disorder Identification Test - Consumption
EMR: Electronic Medical Records
HIV: Human immunodeficiency virus
MSM: Men who have sex with men
PHQ-9: Patient Health Questionnaire-9
PHQ-A: Patient Health Questionnaire- Anxiety
PLWH: People living with HIV
PRO: Patient-Reported Outcome
SD: Standard deviation
UAB: University of Alabama at Birmingham

## References

[1] WHO: https://www.who.int/news-room/fact-sheets/detail/tobacco. Accessed 27 Jan 2018.

[2] CDC: https://www.cdc.gov/tobacco/data_statistics/fact_sheets/adult_data/cig_smoking/index.htm. Accessed 27 Jan 2018.

[3] Mokdad AH, Marks JS, Stroup DF, Gerberding JL (2004). Actual causes of death in the United States, 2000. Jama.

[4] Zyambo CM, Willig JH, Cropsey KL, Carson AP, Wilson C, Tamhane AR, Westfall AO, Burkholder GA. Factors Associated With Smoking Status among HIV-Positive Patients in Routine Clinical Care. J AIDS Clin Res. 2015;6(7):480. 10.4172/2155-6113.1000480 PMC4707973 26767146

[5] Mdodo R, Frazier EL, Dube SR, Mattson CL, Sutton MY, Brooks JT, Skarbinski J (2015). Cigarette smoking prevalence among adults with HIV compared with the general adult population in the United States: cross-sectional surveys. Ann Intern Med.

[6] Helleberg M, Gerstoft J, Afzal S, Kronborg G, Larsen CS, Pedersen C, Bojesen SE, Nordestgaard BG, Obel N (2014). Risk of cancer among HIV-infected individuals compared to the background population: impact of smoking and HIV. Aids.

[7] Drach L, Holbert T, Maher J, Fox V, Schubert S, Saddler LC (2010). Integrating smoking cessation into HIV care. AIDS Patient Care STDs.

[8] Lifson AR, Neuhaus J, Arribas JR, van den Berg-Wolf M, Labriola AM, Read TR, Group ISS (2010). Smoking-related health risks among persons with HIV in the strategies for Management of Antiretroviral Therapy clinical trial. Am J Public Health.

[9] Mamary EM, Bahrs D, Martinez S (2002). Cigarette smoking and the desire to quit among individuals living with HIV. AIDS Patient Care STDs.

[10] CDC (2017). Quitting smoking among adults—United States, 2000–2015. MMWR Morb Mortal Wkly Rep.

[11] Shuter J, Bernstein SL, Moadel AB (2012). Cigarette smoking behaviors and beliefs in persons living with HIV/AIDS. Am J Health Behav.

[12] Pacek LR, Latkin C, Crum RM, Stuart EA, Knowlton AR (2014). Interest in quitting and lifetime quit attempts among smokers living with HIV infection. Drug Alcohol Depend.

[13] Gritz ER, Danysh HE, Fletcher FE, Tami-Maury I, Fingeret MC, King RM, Arduino RC, Vidrine DJ (2013). Long-term outcomes of a cell phone-delivered intervention for smokers living with HIV/AIDS. Clin Infect Dis.

[14] Babb S, Malarcher A, Schauer G, Asman K, Jamal A (2017). Quitting smoking among adults - United States, 2000-2015. MMWR Morb Mortal Wkly Rep.

[15] CDC: https://www.cdc.gov/mmwr/preview/mmwrhtml/mm6044a2.htm [Accessed 20 Mar 2018].

[16] Piasecki TM (2006). Relapse to smoking. Clin Psychol Rev.

[17] Yi Z, Mayorga ME, Hassmiller Lich K, Pearson JL (2017). Changes in cigarette smoking initiation, cessation, and relapse among U.S. adults: a comparison of two longitudinal samples. Tob Induc Dis.

[18] Schafer J, Young J, Bernasconi E, Ledergerber B, Nicca D, Calmy A, Cavassini M, Furrer H, Battegay M, Bucher H (2015). Predicting smoking cessation and its relapse in HIV-infected patients: the Swiss HIV cohort study. HIV Med.

[19] Fiore MCJC, Baker TB (2008). Treating Tobacco Use and Dependence: 2008 Update—Clinical Practice Guidelines.

[20] Services USDoHaH (2010). How Tobacco Smoke Causes Disease: The Biology and Behavioral Basis for Smoking-Attributable Disease: A Report of the Surgeon General.

[21] Fagan P, Augustson E, Backinger CL, O'Connell ME, Vollinger RE, Kaufman A, Gibson JT (2007). Quit attempts and intention to quit cigarette smoking among young adults in the United States. Am J Public Health.

[22] Helvig TM, Sobell LC, Sobell MB, Simco ER (2006). Smokers' narrative accounts of quit attempts: AIDS and impediments to success. Psychol Addict Behav.

[23] Hughes JR, Solomon LJ, Fingar JR, Naud S, Helzer JE, Callas PW (2013). The natural history of efforts to stop smoking: a prospective cohort study. Drug Alcohol Depend.

[24] Hughes JR, Peters EN, Naud S (2008). Relapse to smoking after 1 year of abstinence: a meta-analysis. Addict Behav.

[25] Tesoriero JM, Gieryic SM, Carrascal A, Lavigne HE (2010). Smoking among HIV positive new Yorkers: prevalence, frequency, and opportunities for cessation. AIDS Behav.

[26] Burkhalter JE, Springer CM, Chhabra R, Ostroff JS, Rapkin BD (2005). Tobacco use and readiness to quit smoking in low-income HIV-infected persons. Nicotine Tob Res.

[27] Stanton CA, Moadel AB, Kim RS, Weinberger AH, Shuter J (2015). Loneliness in HIV-infected smokers. AIDS Care.

[28] Yong Hua-Hie, Borland Ron, Cummings K. Michael, Partos Timea (2018). Do predictors of smoking relapse change as a function of duration of abstinence? Findings from the United States, Canada, United Kingdom and Australia. Addiction.

[29] Caraballo RS, Kruger J, Asman K, Pederson L, Widome R, Kiefe CI, Hitsman B, Jacobs DR (2014). Relapse among cigarette smokers: the CARDIA longitudinal study - 1985-2011. Addict Behav.

[30] Zyambo CM, Burkholder GA, Cropsey KL, Willig JH, Wilson CM, Gakumo CA, Westfall AO, Hendricks PS (2019). Predictors of smoking cessation among people living with HIV receiving routine clinical care. AIDS Care.

[31] Kozak MS, Mugavero MJ, Ye J, Aban I, Lawrence ST, Nevin CR, Raper JL, McCullumsmith C, Schumacher JE, Crane HM (2012). Patient reported outcomes in routine care: advancing data capture for HIV cohort research. Clin Infect Dis.

[32] Gilpin EA, Pierce JP, Farkas AJ (1997). Duration of smoking abstinence and success in quitting. J Natl Cancer Inst.

[33] Bing EG, Burnam MA, Longshore D, Fleishman JA, Sherbourne CD, London AS, Turner BJ, Eggan F, Beckman R, Vitiello B (2001). Psychiatric disorders and drug use among human immunodeficiency virus-infected adults in the United States. Arch Gen Psychiatry.

[34] Catalan J, Meadows J, Douzenis A (2000). The changing pattern of mental health problems in HIV infection: the view from London, UK. AIDS Care.

[35] Hughes JR (2007). Effects of abstinence from tobacco: valid symptoms and time course. Nicotine Tob Res.

[36] Balbani AP, Montovani JC (2005). Methods for smoking cessation and treatment of nicotine dependence. Braz J Otorhinolaryngol.

[37] Gilbert DG, McClernon FJ, Rabinovich NE, Plath LC, Masson CL, Anderson AE, Sly KF (2002). Mood disturbance fails to resolve across 31 days of cigarette abstinence in women. J Consult Clin Psychol.

[38] Hughes JR (1992). Tobacco withdrawal in self-quitters. J Consult Clin Psychol.

[39] Ward MM, Swan GE, Jack LM (2001). Self-reported abstinence effects in the first month after smoking cessation. Addict Behav.

[40] Kinnunen T, Korhonen T, Garvey AJ (2008). Role of nicotine gum and pretreatment depressive symptoms in smoking cessation: twelve-month results of a randomized placebo controlled trial. Int J Psychiatry Med.

[41] Shiffman S, Waters AJ (2004). Negative affect and smoking lapses: a prospective analysis. J Consult Clin Psychol.

[42] Zvolensky MJ, Bakhshaie J, Sheffer C, Perez A, Goodwin RD (2015). Major depressive disorder and smoking relapse among adults in the United States: a 10-year, prospective investigation. Psychiatry Res.

[43] Bold KW, McCarthy DE, Minami H, Yeh VM, Chapman GB, Waters AJ (2016). Independent and interactive effects of real-time risk factors on later temptations and lapses among smokers trying to quit. Drug Alcohol Depend.

[44] Kahler CW, Spillane NS, Metrik J (2010). Alcohol use and initial smoking lapses among heavy drinkers in smoking cessation treatment. Nicotine Tob Res.

[45] Rodriguez-Cano R, Lopez-Duran A, Martinez-Vispo C, Martinez U, Fernandez Del Rio E, Becona E (2016). Hazardous alcohol drinking as predictor of smoking relapse (3-, 6-, and 12-months follow-up) by gender. J Subst Abus Treat.

[46] Reynolds NR (2009). Cigarette smoking and HIV: more evidence for action. AIDS Educ Prev.

[47] Bangsberg DR, Deeks SG (2002). Is average adherence to HIV antiretroviral therapy enough?. J Gen Intern Med.

[48] Levine AJ, Hinkin CH, Marion S, Keuning A, Castellon SA, Lam MM, Robinet M, Longshore D, Newton T, Myers H (2006). Adherence to antiretroviral medications in HIV: differences in data collected via self-report and electronic monitoring. Health Psychol.

[49] Reynolds NR (2004). Adherence to antiretroviral therapies: state of the science. Curr HIV Res.

[50] King RM, Vidrine DJ, Danysh HE, Fletcher FE, McCurdy S, Arduino RC, Gritz ER (2012). Factors associated with nonadherence to antiretroviral therapy in HIV-positive smokers. AIDS Patient Care STDs.

[51] O'Cleirigh C, Valentine SE, Pinkston M, Herman D, Bedoya CA, Gordon JR, Safren SA (2015). The unique challenges facing HIV-positive patients who smoke cigarettes: HIV viremia, ART adherence, engagement in HIV care, and concurrent substance use. AIDS Behav.

[52] Shuter J, Bernstein SL (2008). Cigarette smoking is an independent predictor of nonadherence in HIV-infected individuals receiving highly active antiretroviral therapy. Nicotine Tob Res.

[53] Aggarwal B, Mosca L (2010). Lifestyle and psychosocial risk factors predict non-adherence to medication. Ann Behav Med.

[54] Kissinger P, Rice J, Farley T, Trim S, Jewitt K, Margavio V, Martin DH (1999). Application of computer-assisted interviews to sexual behavior research. Am J Epidemiol.

